# MUC1-targeted CAR-T cell secreted anti-PD-1 IgG antibody enhances antitumor activity in Cholangiocarcinoma

**DOI:** 10.1038/s41598-026-49988-w

**Published:** 2026-04-21

**Authors:** Nattarika Khuisangeam, Thanyavi Chinsuwan, Thananya Intanachai, Rattapoom Thaiwong, Chatikorn Boonkrai, Tanapati Phakham, Trairak Pisitkun, Koramit Suppipat, Nattiya Hirankarn, Supannikar Tawinwung

**Affiliations:** 1 Medical Microbiology, Interdisciplinary and International Program, Graduate School, Chulalongkorn University, Bangkok, Thailand; 2 Center of Excellence in Cellular Immunotherapy, Chulalongkorn University, Bangkok, Thailand; 3 Department of Microbiology, Faculty of Medicine, Chulalongkorn University, Bangkok, Thailand; 4 Chulalongkorn Comprehensive Cancer Center, King Chulalongkorn Memorial Hospital, Bangkok, Thailand; 5 Center of Excellence in Systems Biology, Faculty of Medicine, Chulalongkorn University, Bangkok, Thailand; 6 Department of Research Affairs, Faculty of Medicine, Chulalongkorn University, Bangkok, Thailand; 7 Center of Excellence in Immunology and Immune-mediated Diseases, Department of Microbiology, Faculty of Medicine, Chulalongkorn University, Bangkok, Thailand; 8 Department of Pharmacology and Physiology, Faculty of Pharmaceutical Sciences, Chulalongkorn University, Bangkok, 10330 Thailand

**Keywords:** Chimeric antigen receptor (CAR) T cells, Mucin-1 (MUC1), PD-1 blockade, Cholangiocarcinoma (CCA), Armored CAR, Cancer, Immunology, Oncology

## Abstract

Cholangiocarcinoma (CCA) is a highly aggressive malignancy with poor prognosis and limited therapeutic options. Gene expression analysis of patient samples and flow cytometry of CCA cell lines demonstrated significant upregulation of MUC1 and PD-L1, supporting their relevance as immunotherapeutic targets. To address immune suppression mediated by PD-1/PD-L1 signaling, we developed MUC1-specific CAR-T cells engineered to secrete a full-length anti-PD-1 immunoglobulin (MUC1.PD1 CAR-T cells). The MUC1.PD1 CAR-T cells exhibited high transduction efficiency and showed memory phenotypes comparable to conventional MUC1 CAR-T cells. Both constructs mediated antigen-specific cytotoxicity against MUC1⁺PD-L1⁺ CCA cells in vitro. Under chronic antigen stimulation, MUC1.PD1 CAR-T cells displayed reduced detectable PD-1 expression and maintained superior proliferative capacity compared with conventional MUC1 CAR-T cells. In vivo, in a NOD/SCID xenograft model of CCA, conventional MUC1 CAR-T cells failed to control tumor growth, whereas MUC1.PD1 CAR-T cells significantly suppressed tumor progression. These findings demonstrate that the secretion of full-length anti-PD-1 immunoglobulin enhances CAR-T cell function and anti-tumor activity in the context of CCA.

**Supplementary Information:**

The online version contains supplementary material available at 10.1038/s41598-026-49988-w.

## Introduction

Cholangiocarcinoma (CCA) exhibits striking geographic variation, with the highest incidence reported in regions endemic for liver fluke infections. In Northeastern Thailand, Laos, and Vietnam, infection with *Opisthorchis viverrini*, and in China, Taiwan, South Korea, and the Russian Far East, infection with *Clonorchis sinensis*, significantly increase CCA risk compared to global levels^1–3^. CCA is classified according to anatomical location as intrahepatic (iCCA), perihilar (pCCA, Klatskin tumor), or distal CCA (dCCA). This classification is clinically important, as it informs diagnosis, treatment strategies, and prognosis^4^. In Thailand, most patients are diagnosed with stage IV disease, and survival outcomes remain poor. For iCCA, cumulative survival following surgical resection is 52.1% at one year, 21.7% at three years, and only 11.2% at five years, with a median survival of 12.4 months^5–7^. These statistics highlight the urgent need for more effective therapies.

Adoptive cell therapy with chimeric antigen receptor (CAR) T cells has demonstrated unprecedented efficacy in hematologic malignancies^8^. Translating this success to solid tumors such as CCA remains challenging due to tumor-associated heterogeneity and the immunosuppressive tumor microenvironment (TME). Among potential targets, Mucin 1 (MUC1), a type I transmembrane glycoprotein normally expressed on the apical surface of epithelial cells, is aberrantly overexpressed in a hypoglycosylated form (tumor-associated MUC1; tMUC1) across multiple cancers, including CCA^9–12^. Elevated tMUC1 levels in CCA have been associated with tumor invasiveness, immune evasion, and poor prognosis^13,14^. Preclinical studies have also shown that MUC1-targeted CAR-T cells exhibit antigen-specific antitumor activity in solid tumors including breast cancer^15^ and CCA^16,17^.

However, CAR-T cell therapy in solid tumors is hindered by immune suppression within the TME, notably through upregulation of programmed death-ligand 1 (PD-L1)^18–20^. Blockade of the PD-1/PD-L1 axis has demonstrated clinical benefit in advanced CCA, as shown in two pivotal phase III trials: TOPAZ-1, where durvalumab plus gemcitabine–cisplatin improved outcomes^21^, and KEYNOTE-966, where pembrolizumab plus chemotherapy yielded similar benefits^22^. These findings underscore the therapeutic relevance of targeting PD-1/PD-L1 in CCA.

In the present study, we developed CAR-T cells directed against tMUC1 and engineered them to secrete an anti-PD-1 antibody (MUC1.PD1 CAR-T). Here, we report on the generation and preclinical evaluation of armored MUC1 CAR-T cells secreting anti-PD-1 Ig as alternative therapeutic approach for CCA.

## Materials and methods

### Gene expression and survival analysis of *MUC1* and *PD-L1/CD274*

The expression levels of *MUC1* and *PD-L1/CD274* were analyzed across primary tumor samples (*n* = 215) and matched adjacent normal tissues (*n* = 180) from patients with cholangiocarcinoma (CCA) and pancreatic adenocarcinoma (PAAD). Gene expression data were obtained using the Gene Expression Profiling Interactive Analysis 2 (GEPIA2) platform (http://gepia2.cancer-pku.cn). The data were normalized and transformed to a log2 scale using formula log2(TPM + 0.001), where TPM refers to transcripts per million and a pseudocount of 0.001 was added to prevent undefined values from zero expression. Differential expression analysis between tumor and normal tissues was performed using a paired t-test, with statistical significance set at *p* < 0.05.

Survival analysis was conducted using the GEPIA2 platform which integrates RNA sequencing expression data from The Cancer Genome Atlas (TCGA) and Genotype-Tissue Expression (GTEx) projects. The prognostic significance of *MUC1* and *PD-L1/CD274* expressions were assessed in patients with CCA and PAAD. Patients were stratified into high- and low-expression groups based on the median expression level of each gene. Kaplan–Meier survival curves were generated, and statistical differences between groups were evaluated using the log-rank (Mantel–Cox) test. Chi-square (χ²) values and corresponding *p*-values were reported, with statistical significance set at *p* < 0.05. Both overall survival (OS) and disease-free survival (DFS) analyses were performed.

### Cell culture

Cholangiocarcinoma (CCA) cell lines HuCCT-1 and KKU-213, along with the human embryonic kidney cell line 293 T, were obtained from the American Type Culture Collection (ATCC). HuCCT-1 cells were cultured in Roswell Park Memorial Institute (RPMI)−1640 medium (Gibco, Thermo Fisher Scientific, Waltham, MA, USA). KKU-213 and 293 T cells were maintained in Dulbecco’s Modified Eagle Medium (DMEM) (HyClone, Cytiva, South Logan, UT, USA). All cell lines were cultured at 37 °C in a humidified atmosphere containing 5% CO₂ and were routinely screened to ensure the absence of mycoplasma contamination.

### Plasmid construction

The anti-PD-1 immunoglobulin (Ig) antibody used in this study is derived from the monoclonal antibody Nivolumab (Drugbank accession number: DB09035). To optimize the expression of this antibody in human cells, codon optimization was performed using an online tool, Gene Designer 2.0. The synthesized sequences were then produced by GenScript (GenScript, NJ, USA). To facilitate the detection of transduction efficiency, the anti-PD-1 Ig antibody was cloned into an SFG-based retroviral vector, which also contains a truncated CD19 (dCD19) marker.

### PBMC isolation and T cell activation

Peripheral blood samples were collected from healthy donors following protocols approved by the Institutional Review Board of the Faculty of Medicine, Chulalongkorn University (IRB No. 437/62). Written informed consent was obtained from all subjects, and the study was conducted in accordance with relevant guidelines and regulations. Peripheral Blood Mononuclear Cells (PBMCs) were isolated using Ficoll-Paque Premium (Cytiva, Uppsala, Sweden). Subsequently, 1 × 10^6^ PBMCs were activated on plates coated with 1 µg/mL anti-CD3 (clone OKT3, Miltenyi Biotec, Bergisch Gladbach, Germany) and 1 µg/mL purified anti-CD28 antibody (BD Biosciences, Franklin Lakes, NJ, USA) for 72 h in the presence of recombinant human interleukin-2 (rhIL-2) at final concentration of 50 U/mL.

### Production of retrovirus and T cell transduction

Gamma (γ)-retrovirus supernatants were produced as previously described^23^. Briefly, 293 T cells were co-transfected with the SFG plasmid containing the MMLV promoter encoding either the CAR transgene or anti-PD-1 Ig, along with a MoMLV Gag-Pol plasmid and the RD114 envelope plasmid, using GeneJuice transfection reagent (Novagen, Billerica, MA, USA). Retroviral supernatants were harvested at 48 and 72 h post-transfection and subsequently stored at − 80 °C until further use.

For T cell transduction, the retroviral supernatant was added to a 24-well non-tissue culture-treated plate coated with recombinant fibronectin fragment (FN CH-296; RetroNectin; Takara Bio, Shiga, Japan) and centrifuged at 2000 × g for 90 min. After centrifugation, the retroviral supernatant was removed and activated T cells (0.2 × 10^6^/well) in cultured media supplemented with IL-7 (10 ng/ml) and IL-15 (5 ng/ml) were added. The plates were centrifuged at 400×g for 5 min, and the cells were left to expand in culture for further experiments. To generate MUC1 CAR-T cells secreting anti–PD-1 Ig, sequential transductions were performed. T cells were first transduced with retroviral supernatant encoding the MUC1 CAR. The following day, the cells were harvested and transduced with retrovirus encoding anti–PD-1 Ig as mentioned above. For Non‑transduced (NT) control T cells, activated T cells were added to a well pre-coated with RetroNectin without viral supernatant.

### Flow cytometry

The antibodies used in this study are as follows. PerCP anti-human CD3 (clone UCHT1, cat: 560835; BD Biosciences), PE anti-human CD8 (clone SK1, cat: 340046; BD Biosciences), FITC mouse IgM, κ Isotype Control (clone G155228, cat: 564680; BD Biosciences), FITC anti-human CD4 (clone RPA-T4, cat: 564419; BD Biosciences), FITC anti-human TIM-3 (CD366) (clone 7D3, cat: 565568; BD Biosciences), VioBlue mouse IgG2b, κ Isotype Control (clone 27–35, cat: 562748; BD Biosciences), VioBlue CD62L (clone DREG-56, cat: 304828; Biolegend), VioGreen CD45RO (clone UCHL1, cat: 304246; Biolegend), VioBlue anti-human LAG-3 (CD223) (clone T47-530, cat: 565720; BD Biosciences), VioGreen IgG2a, κ Isotype Control (clone MOPC-173, cat: 563483; BD Biosciences), VioGreen anti-human TIGIT (clone 741182, cat: 747482; BD Biosciences), PE-Cy7 Mouse IgG1 κ Isotype Control (clone MOPC-21, cat: 565573; BD Biosciences), PE-Cy7 anti-human CD279 (PD-1) (clone EH12.1, cat: 561272; BD Biosciences). APC anti-human CD19 (clone HIB19, cat: 555415; BD Biosciences) and 7-AAD (cat: 559 925BD; Biosciences).

To assess CAR transduction efficiency, cells were stained with Alexa Fluor^®^ 488-conjugated goat anti-human IgG (H + L) antibody (cat: 109–605-003; Jackson ImmunoResearch) for 15 min at 4 °C to detect the human IgG CH3 domain in the CAR spacer region as previously described^24^. Flow cytometric analysis was conducted using the MACSQuant^®^ Analyzer 10 (Miltenyi Biotec), and data were processed and analyzed using FlowJo software, version 10.7.1 (BD Biosciences).

### Enzyme-linked immunosorbent assay (ELISA)

To evaluate the binding of secreted anti-PD-1 Ig, culture supernatant from CAR-T cells was collected on day 4 following transduction and stored at −20 °C. In brief, 96 well ELISA plates are coated with Human PD-1/PDCD1 Protein, His-tag (ACROBiosystems, cat# PD1-H522a) at 50 ng per well in 100 µl PBS at 4 °C overnight. Plates were washed 3 times with PBS containing 0.05% Tween-20 (PBST) before adding 200 µL of culture supernatant, followed by two-fold serial dilutions. After incubation at 37 °C for 1 h, wells were washed and incubated with HRP-conjugated goat anti-human IgG Fc antibody (Invitrogen, cat# A55745; 1:8,000 dilution in PBST) for 1 h at 37 °C. Plates were washed and developed with 100 µL SIGMAFAST OPD substrate (Sigma-Aldrich) for 20 min at room temperature in the dark. Reactions were stopped with 50 µL 2 N sulfuric acid, and absorbance was measured at 492 nm.

### Western blot analysis

To detect secreted anti-PD-1 Ig protein, Western blot analysis was performed. 293 T cells were transduced with Gamma (γ)-retrovirus encoding anti-PD-1 Ig and the culture supernatant was collected. Briefly, equal volumes (30 µL) of cell culture supernatants were subjected to electrophoresis on a 10% SDS-PAGE gel for protein separation based on molecular weight. Proteins were subsequently transferred onto an Immuno-Blot PVDF membrane (Bio-Rad). Membranes were blocked with 5% skim milk in PBS, followed by washing with PBS containing 0.1% Tween-20 (PBST). Membranes were then incubated with a horseradish peroxidase (HRP)-conjugated goat anti-human IgG Fc antibody (Invitrogen, Cat# A55745) diluted 1:8,000 in blocking buffer. Protein bands were visualized using Clarity™ and Clarity Max™ ECL Western Blotting Substrates (Bio-Rad), enabling chemiluminescent detection of the target proteins.

### Co-culture experiment

The anti-tumor activity of CAR-T cells was evaluated by co-culturing them with cholangiocarcinoma cell lines (HuCCT-1 and KKU-213) as target cells, and 293 T cells as a negative control. Co-cultures were established at effector-to-target (E: T) ratios of 1:2, 1:1, 2:1, and 4:1 in DMEM supplemented with 10% fetal bovine serum (FBS) and 2 mM L-GlutaMAX, in the absence of exogenous cytokines, and incubated for 72 h. Following incubation, cells were harvested and stained with 7-AAD (BD Biosciences) to identify dead cells and PE-conjugated anti-CD3 antibody to label T cells. The number of viable target cells and effector cells were counted using the Accuri C6 flow cytometer with fixed acquisition volume. The percentage of tumor cell inhibition was calculated using the following formula:

$$\% {\text{Inhibition }} = {\text{ }}100{\text{ }} - {\text{ }}(\frac{{{\text{number of Target (experiment)}}}}{{{\text{number of Target (Target alone)}}}} \times 100)$$

### Repeated antigen stimulation assay

CAR-T cells were co-cultured with HuCCT-1 cholangiocarcinoma cells at an effector-to-target (E: T) ratio of 1:1 (5,000:5,000) in the presence of recombinant human interleukin-2 (IL-2) at a final concentration of 25 U/mL. After 72 h, T cells were harvested and restimulated with freshly seeded HuCCT-1 cells for total of three 72-h cycles. The number of residual effector cells and the expression of exhaustion markers, including PD-1, TIM-3, TIGIT, and LAG-3 were evaluated at each round.

### Animal study

All procedures were reviewed and approved by the Chulalongkorn University Animal Care and Use Committee, under protocol No. 23–33-008 and conducted in accordance with relevant guidelines and regulations. The study is reported in adherence to ARRIVE guidelines. An experiment was performed on female NOD/SCID mice (6–8 weeks old, *n* = 5 per group, total *n* = 15) weighing 16–20 g, purchased from Nomura Siam International (Bangkok, Thailand). Mice were engrafted subcutaneously (s.c.) in the right flank with 1 × 10^6^ HuCCT-1 cells mixed at a 1:1 ratio with Matrigel^®^ Basement Membrane Matrix (Corning, USA, Cat#356234) to facilitate tumor engraftment. When tumors reached approximately 100 mm³, mice were randomly assigned into three treatment groups, and administered 1.5 × 10^7^ cells of NT, MUC1, or MUC1.PD1 CAR-T cells intravenously (i.v.) for two doses. Blood samples were collected weekly. Tumor growth was monitored by caliper measurement on a weekly basis. Body weight was also measured weekly as a general health indicator. Anesthesia was administered using 100% isoflurane via inhalation with the open-drop method during tumor measurement. Mice showing visible signs of distress or reaching a tumor diameter of 12 mm (in either length or width) were euthanized in accordance with institutional animal care and use guidelines, using CO₂ inhalation (30–70% volume per minute) followed by cervical dislocation. Tumor volume was calculated using the following formula:

$$\text{Tumor volume} =\:\frac{\mathrm{l}\mathrm{e}\mathrm{n}\mathrm{g}\mathrm{t}\mathrm{h}\:\:\mathrm{x}\:\:{\mathrm{w}\mathrm{i}\mathrm{d}\mathrm{t}\mathrm{h}}^{2}}{2}$$

### Statistical analysis

All data are presented as the mean ± standard error of the mean (S.E.M). Statistical analyses were performed using GraphPad Prism version 8.0 (GraphPad Software, Boston, MA, USA). Differences between or among groups were evaluated using one-way or two-way analysis of variance (ANOVA), as appropriate for the experimental design. Post hoc analysis was performed using Tukey’s multiple comparisons test to identify statistically significant differences between individual group means. For survival analysis, survival curve was estimated using the Kaplan–Meier method, and statistical differences between groups were determined using the log-rank (Mantel–Cox) test. Where applicable, Chi-square (χ²) tests were used to assess the significance of categorical distributions. Tumor growth over time was analyzed using a linear mixed-effects model with restricted maximum likelihood estimation. Post hoc comparisons were performed using the Holm–Šídák method, comparing least-squares means of the control group with those of the MUC1 and MUC1.PD1 groups. A *p-value* of less than 0.05 (*p* < 0.05) was considered indicative of statistical significance.

## Results

### Expressions of *MUC1* and *PD-L1* (*CD274*) and their associations with survival in patients with cholangiocarcinoma

**Fig. 1:**
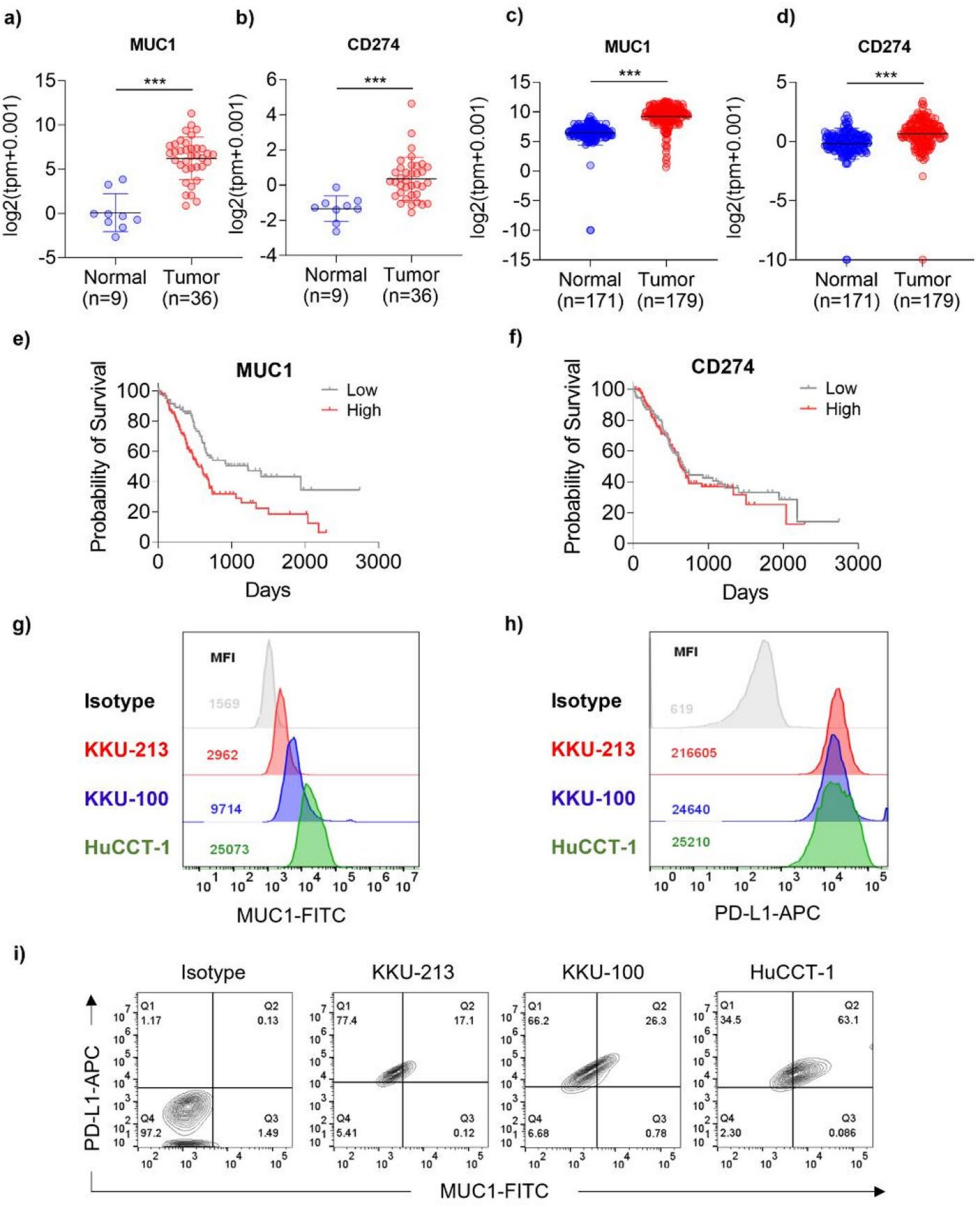
Expressions of MUC1 and PD-L1 (CD274) on cholangiocarcinoma (CCA) and pancreatic adenocarcinoma (PAAD) and their associations with survival in patients. (a–d) mRNA expression levels of MUC1 and CD274 in cholangiocarcinoma **(a-b)** (*n* = 36 tumors, *n* = 9 normal) and pancreatic adenocarcinoma **(c-d)** (*n* = 179 tumors, *n* = 171 normal). RNA sequencing data were obtained from The Cancer Genome Atlas (TCGA) and Genotype-Tissue Expression (GTEx) databases, and expression values are presented as log2-transformed transcript per million (TPM + 0.001) to ensure normalization and comparability across samples. Data are represented with individual dot plots. Unpaired t-test, ***p < 0.001. **(e**,** f)** Kaplan–Meier survival curves illustrating the relationship between high versus low expression of MUC1 (e) and PD-L1(f) and overall survival outcomes in patients with cholangiocarcinoma and pancreatic adenocarcinoma. Statistical significance was determined using the log-rank test and Mantel-Cox method,, *p* < 0.001 for MUC1, *p* = 0.639 for PD-L1. **(g**,** h)** Flow cytometric quantification of surface expression of MUC1 (g) and PD-L1 (h) proteins across multiple human CCA cell lines **(i)** Co-expression analysis of PD-L1 and MUC1 on CCA cell lines (KKU-213, KKU-100, and HuCCT-1).

We first evaluated *MUC1* and *CD274* gene expression in cholangiocarcinoma (CCA) and pancreatic adenocarcinoma (PAAD). RNA-Seq data from TCGA and GTEX were downloaded from cBioPortal^25^, and transformed to log2-transformed transcripts per million (TPM + 0.001). A total of 36 CCA tumor samples and 9 normal controls, as well as 179 PAAD tumor samples and 171 normal controls, were analyzed. Both MUC1 (Fig. 1a and c) and CD274 (PD-L1) (Fig. 1b and d) were significantly upregulated in tumor tissues compared to normal controls (*p* < 0.001), highlighting their potential relevance as immunotherapeutic targets in CCA and PAAD. Overall survival analysis of CCA and PAAD data revealed that lower survival was associated with “High MUC1+” (*n* = 130; log2(TPM + 0.001) $\:\ge\:$ 8.73) compared to “low MUC1+” (*n*= 85) (Fig. 1e, *p* = 0.001). However, no significant difference in overall survival was observed between patients with high PD-L1 (*n* = 98; log2(TPM+0.001) $\:\ge\:$ 0.608) and low PD-L1 expression (*n*=117) (Fig. 1f).

We next evaluated surface protein expression of MUC1 and PD-L1 proteins in CCA cell lines, including KKU-100, KKU-213, and HuCCT-1, using flow cytometry. MUC1 was expressed across all tested CCA cell lines at differential levels (Fig. 1g). while PD-L1 was expressed at a high level on all of them (Fig. 1h). We also showed that PD-L1 and MUC1 double positivity on these cell lines varied accordingly, but double negative cells were rare (Fig. 1i). Therefore, these results validate MUC1 and PD-L1 as potential target antigens for immunotherapy using CAR-T cells.

### Generation of CAR-T cells targeting MUC1 with self-release anti-PD-1 Ig

**Fig. 2:**
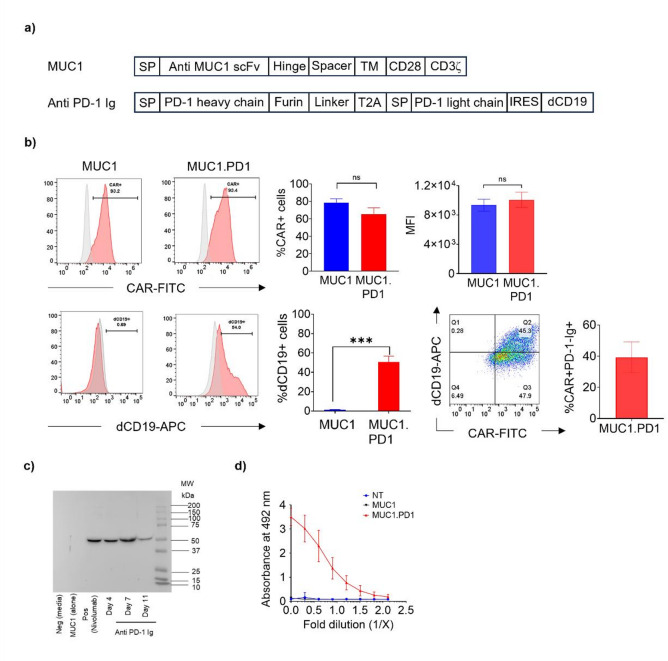
Generation of CAR-T cells targeting MUC1 armored with anti-PD-1 Ig. **(a)** Schematic representation of vector maps for MUC1 CAR construct and the anti-PD-1 immunoglobulin. The truncated CD19 (dCD19) was included as a transduction control marker. **(b)** Representative flow cytometry plots and quantification showing CAR and dCD19 expression on day 4 post-transduction and the frequency of CAR⁺/anti–PD-1-Ig⁺ double-positive cells. Data are shown as mean ± S.E.M., *n* = 6 donors per group; one-way ANOVA with Tukey’s test; *p < 0.05; ns, not significant. **(c)** Western blot analysis of culture supernatants from 293 T packaging cells transduced with the anti-PD-1 Ig construct, probed with goat anti-Fc HRP-conjugated antibody. Original blot is presented in Supplementary Figure S1. **(d)** ELISA demonstrates specific binding of anti-PD-1 immunoglobulin secreted by CAR-T cells to recombinant human PD-1 protein. Bound immunoglobulin was detected using an HRP-conjugated anti-human IgG secondary antibody and OPD substrate.

To generate CAR-T cells targeting tumor-associated MUC1 (tMUC1), the single-chain variable fragment (scFv) derived from the previously developed mAb clone HMFG2^23^, specific to an epitope within the tandem repeat core protein of tMUC1, was used. The scFv was fused to an IgG2-derived CH3 spacer, followed by the CD28 transmembrane and intracellular signaling domains, and the CD3ζ activation domain, as illustrated in Fig. 2a. To generate CAR-T cells capable of simultaneously targeting MUC1 and blocking PD-1-mediated immunosuppression, the human anti-PD-1 Ig sequence, derived from Nivolumab, was cloned into a separate retroviral vector for co-expression. This strategy produced anti-PD-1 Ig secreted CAR-T cells targeting MUC1 (designated MUC1.PD1). Both the MUC1 CAR-T cells (MUC1) and the dual-expressing MUC1.PD1 CAR-T cells demonstrated high transduction efficiency, with over 80% CAR-positive cells and comparable mean fluorescence intensities (MFIs) at day 4 post-transduction (Fig. 2b). Expression of the anti-PD-1 Ig was monitored indirectly through the transduction control marker dCD19. On day 4, double-transduced CAR+dCD19 + T cells accounted for 41.43 ± 1.15% of the population (Fig. 2c).

To confirm the secretion of anti-PD-1 Ig into the culture media, supernatant from 293 T cell transduced to express anti-PD-1 Ig were collected on day 4, 7, and 11 post-transduction and analyzed by western blot. Culture media alone and supernatants corrected on day 4 from 293 T cell transduced with MUC1 CAR alone were used as negative controls, while Nivolumab served as a positive control. A protein band corresponding to the secreted anti-PD-1 Ig (~ 50 kDa under reducing conditions) was detected in the supernatant (Fig. 2d, Fig. S1). Under non-reducing conditions, a predominant band was observed at approximately 150 kDa, consistent with the fully assembled IgG heterotetramer and comparable to the positive control (nivolumab) (Fig. S2). Furthermore, functional binding of the secreted anti-PD-1 Ig to PD-1 protein was confirmed by ELISA. The supernatant from MUC1.PD1 CAR-T cells, but not from MUC1 CAR-T cells, is bound to recombinant PD-1 in a dose-dependent manner (Fig. 2e), demonstrating effective secretion and binding activity of the secreting antibody.

### Co-expression of anti-PD-1 Ig does not affect MUC1 CAR-T cell expansion, immunophenotype, or cytolytic activity

**Fig. 3:**
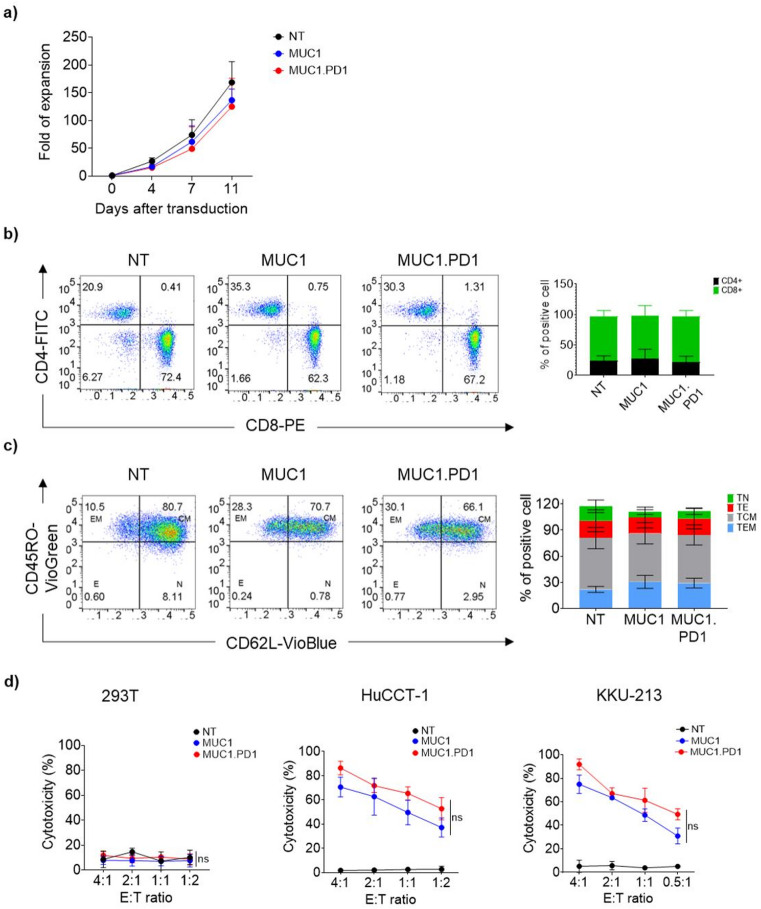
Phenotypic characterization and antigen-specific cytotoxicity of MUC1 and MUC1.PD1 CAR-T cells. **(a)** Fold expansion of total T cells relative to cell counts on day 0. **(b)** Representative flow cytometry plots (left) and summary data (right) of CD4 + and CD8 + T cell subsets on day 11 post-transduction. **(c)** Representative flow cytometry plots (left) and summary data (right) of memory phenotype based on CD62L and CD45RO expression on day 11 post-transduction. Four subsets were defined: central memory (T_CM,_ CD62L+/CD45RO+), effector memory (T_EM,_ CD62L-/CD45RO+), naïve (T_N,_ CD62L+/CD45RO-), and terminal effector (T_CM,_ CD62L-/CD45RO-). Data are presented as mean ± S.E.M., *n* = 6 donors per group; one-way ANOVA with Tukey’s test. **(d)** Cytotoxicity activity of CAR-T cells against HuCCT-1 KKU-213, and 293 T cell lines at E: T ratios of 0.5:1, 1:1, 2:1, and 4:1 for 72 h in the absence of exogenous cytokines. Data are presented as mean ± S.E.M., *n* = 3 donors per group; two-way ANOVA with Tukey’s test; ns, not significant between MUC1 and MUC1.PD1 CAR-T groups.

To determine whether co-expression of anti-PD-1 Ig affects T cell proliferation and phenotype, we analyzed the expansion and immunophenotypic profiles of CAR-T cells following retroviral transduction. Total T cell expansion over the 11-day culture period was comparable among non‑transduced (NT), MUC1 CAR, and MUC1.PD1 CAR-T cells (Fig. 3a). On day 11 post-transduction, flow cytometric analysis of the CD8 + and CD4 + T cell subsets revealed a consistent CD8:CD4 ratio of approximately 4:1 across all groups (Fig. 3b). T cell memory phenotypes were further assessed by evaluating CD62L and CD45RO expression. All three groups exhibited a comparable T cell memory phenotypic profile, with the majority of cells displaying a central memory (T_CM,_ CD62L+/CD45RO+) phenotype, followed by effector memory (T_EM_, CD62L-/CD45RO+) (Fig. 3c), These findings indicates that incorporation of the anti-PD-1 Ig transgene does not alter T cell expansion and immunophenotype of CAR-T cell products.

We next assessed the cytotoxicity of MUC1 and MUC1.PD1 CAR-T cells using a 72-hour in vitro cytotoxicity assay. Two CCA cell lines, KKU-213 and HuCCT-1, both expressing endogenous MUC1 and PD-L1, were utilized as target cells. The 293 T cell line, which lacks expression of MUC1 and PD-L1, served as a negative control. Both MUC1 and MUC1.PD1 CAR-T cells demonstrated dose-dependent cytotoxicity against KKU-213 and HuCCT-1 cells, with no significant cytotoxicity observed against 293 T cells, confirming antigen-specific activity. The cytotoxic efficacy of MUC1.PD1 CAR-T cells was comparable to that of MUC1 CAR-T cells, and both groups exhibited significantly higher tumor cell killing than NT cells (Fig. 3d).

### Effects of secreted anti-PD-1 Ig on exhaustion markers and proliferation of MUC1 CAR-T cells under repeated antigen stimulation in vitro

**Fig. 4:**
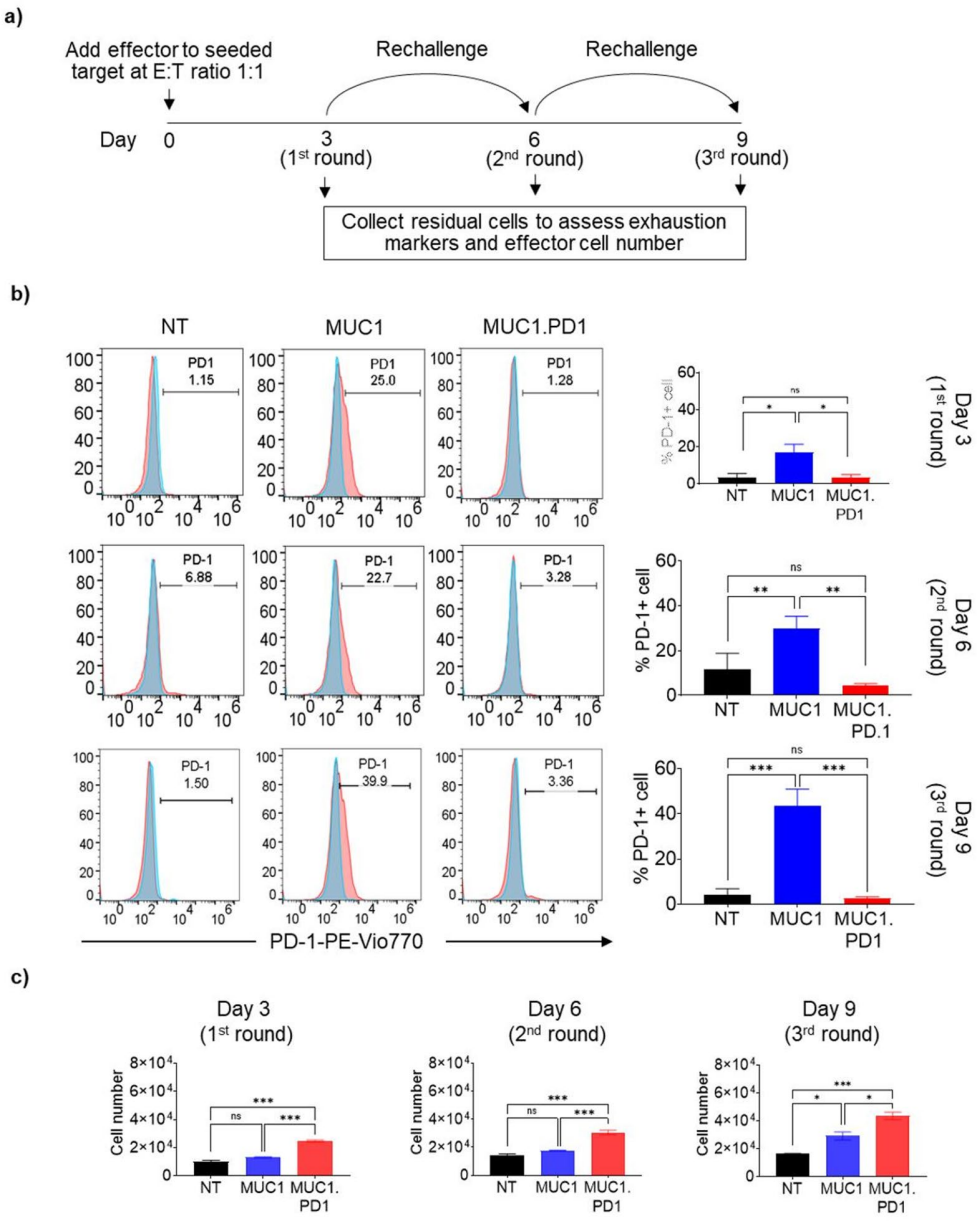
MUC1.PD1 CAR-T cells exhibit reduced PD-1 surface expression and enhanced proliferative capacity following repeated antigen stimulation. **(a)** Schematic timeline illustrating the three repeated rounds of antigen stimulation applied to T cells over the course of the experiment. Each stimulation round involved exposure to antigen, followed by exhaustion markers analysis and cell counting. **(b)** Flow cytometric analysis of PD-1 surface expression on T cells measured immediately after each round of antigen stimulation. **(c)** Quantification of viable effector T cells following each stimulation round using the trypan blue exclusion assay. Data are presented as mean ± S.E.M., *n* = 3 donors per group; one -way ANOVA with Tukey’s multiple comparisons test; *p < 0.05, **p < 0.01, ***p < 0.001.

To assess whether secreted anti-PD-1 Ig rescues CAR-T cells from exhaustion induced by repeated antigen stimulation, we performed a serial co-culture assay. MUC1 CAR-T cells, MUC1.PD1 CAR-T cells, or non‑transduced (NT) T cells were stimulated with HuCCT-1 cells at an effector-to-target (E: T) ratio of 1:1 in the presence of 25 U/mL IL-2 for 72 h. Fresh tumor cells were added every 72 h for a total of three stimulation cycles. At the end of each cycle, T cells were analyzed for proliferation and surface expression of exhaustion markers including PD-1, TIM-3, and LAG-3. The experimental design and timeline are shown in Fig. 4a. Following repeated stimulation with CCA cells, MUC1 CAR-T cells exhibited a significant increase in PD-1 expression compared with NT cells. In contrast, surface PD-1 was minimally detected on MUC1.PD1 CAR-T cells (Fig. 4b). Expression levels of other inhibitory receptors including TIM-3, LAG-3, and TIGIT did not differ significantly between MUC1 and MUC1.PD1 CAR-T cells (Fig. S3). Notably, MUC1.PD1 CAR-T cells exhibited significantly greater expansion compared to MUC1 CAR-T cells after chronic stimulation (Fig. 4c). To determine whether the secreted anti–PD-1 Ig induces transcriptional downregulation of PD-1, PDCD1 mRNA levels were measured during repeated antigen stimulation with HuCCT-1 cells. No significant difference in PDCD1 transcript levels was observed between the two groups (Fig. S4). Together, these results suggest that secretion of anti-PD-1 Ig blocks PD-1 binding and enhances the proliferative capacity of MUC1 CAR-T cells under chronic antigen stimulation.

### MUC1 CAR-T cell with self-release anti-PD-1 Ig enhanced antitumor activity *in vivo*

**Fig. 5:**
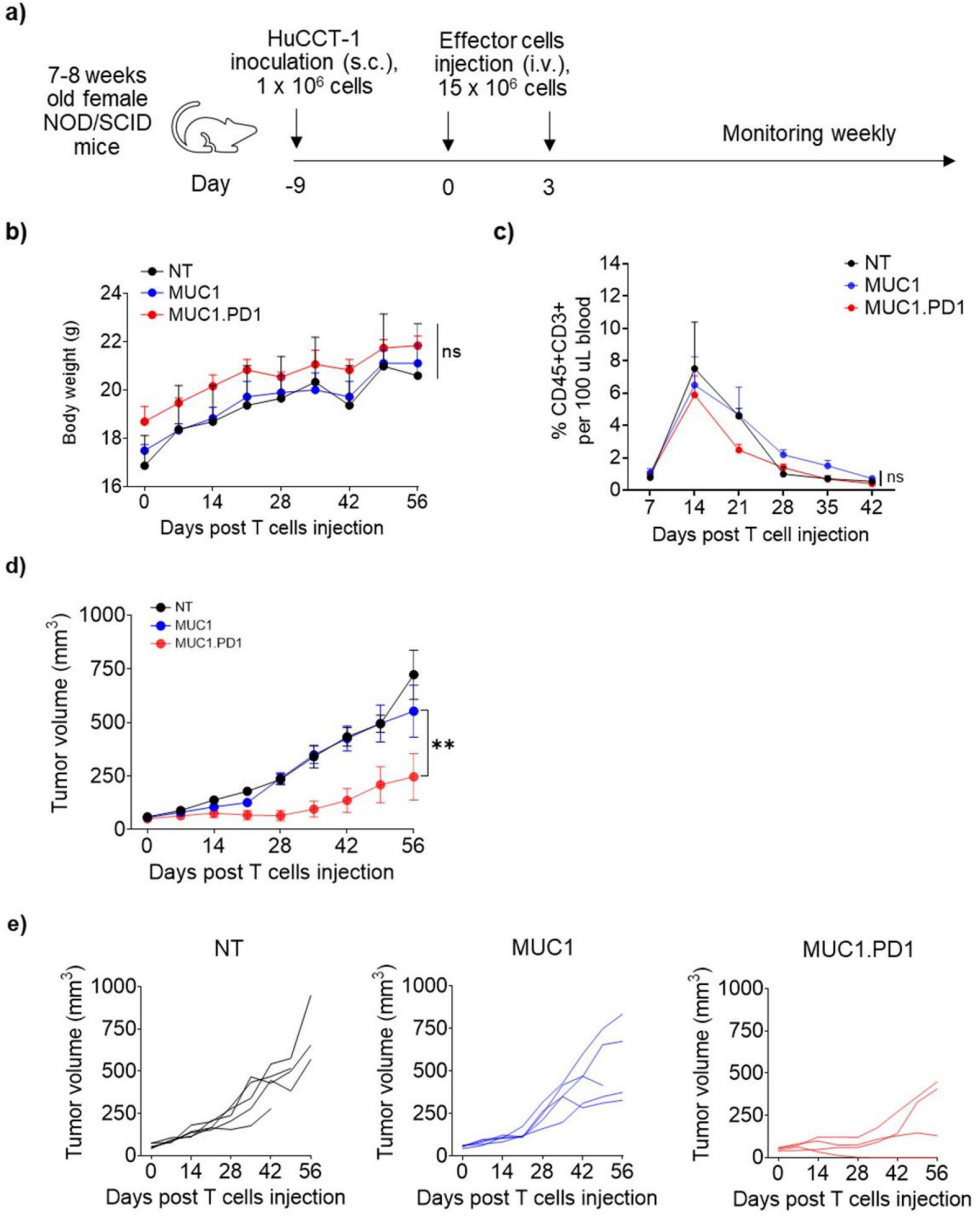
MUC1.PD1 CAR-T cells enhance anti-tumor activity against MUC1 + cholangiocarcinoma in vivo. **(a)** NOD/SCID mice were engrafted subcutaneously in the right flank with 1 × 10⁶ HuCCT-1 CCA cells. Mice were administered intravenously with either NT cells, MUC1 CAR-T cells, or MUC1.PD1 CAR-T cells (1.5 × 10⁷ cells per dose) on day 0 and day 3 (*n* = 5 mice/group, total *n* = 15). Tumor growth was subsequently monitored on a weekly basis **(b)** Body weight of mice was monitored throughout the treatment period to assess general health and treatment toxicity. Data are shown as mean ± S.E.M; two-way ANOVA with Tukey’s multiple comparison test., ns, not significant. **(c)** Frequency of human circulating T cells (CD45⁺CD3⁺) in peripheral blood samples collected post-treatment. Data are shown as mean ± S.E.M; two-way ANOVA with Tukey’s multiple comparison test., ns, not significant. **(d)** Mean tumor volumes over time in each treatment group. Data are presented as mean ± S.E.M. (*n* = 5); linear mixed-effects model by restricted maximum likelihood with Holm-Šídák’s multiple comparisons test **adjusted *p* < 0.01. NT, non-transduced (e) Individual tumor growth curves for each mouse within the treatment groups.

To asses*s in vivo* efficacy, we evaluated MUC1 and MUC1.PD1 CAR-T cells in a cholangiocarcinoma xenograft model. NOD/SCID mice bearing established tumors received two doses of 1.5 × 10⁷ T cells at three-day intervals. Tumor progression was monitored weekly by caliper measurements, and peripheral blood was analyzed for human T cells persistence (Fig. 5a). CAR-T cell administration was well tolerated, with no significant changes in body weight observed across treatment groups (Fig. 5b). Human CD45⁺CD3⁺ T cells were detectable in peripheral blood on day 14 post-infusion but declined thereafter, with no significant differences in persistence among groups (Fig. 5c). Interestingly, treatment with MUC1.PD1 CAR-T cells led to enhanced tumor growth inhibition, demonstrating significantly superior antitumor activity, compared with MUC1 CAR-T cell alone (Fig. 5d and e). These results demonstrate that secretion of anti-PD-1 Ig enhances the therapeutic efficacy of MUC1 CAR-T cells against MUC1⁺PD-L1⁺ cholangiocarcinoma xenografts.

## Discussion

Cholangiocarcinoma (CCA) remains a challenging malignancy with limited therapeutic options, underscoring the need for innovative treatment strategies. In the present study, our RNA analysis revealed significant upregulation of MUC1 in CCA, which is associated with poor prognosis, along with increased expression of CD274, indicating an immunosuppressive tumor microenvironment (TME). Given these findings, we developed a MUC1-targeting CAR-T cell therapy engineered to secrete anti-PD-1 immunoglobulin (Ig) to counteract immune suppression within the TME. Our results demonstrate that this dual-function CAR-T cell specifically lyses CCA cells and blocks PD-1 expression on T cells upon repeated antigen stimulation. Furthermore, CAR MUC1 T cells secreted anti-PD-1 Ig improved tumor control in an in vivo xenograft model of CCA, indicating that intrinsic immune checkpoint inhibition can enhance CAR-T cell function in CCA.

To target tumor-associated MUC1 on CCA cells, the CAR construct utilizes the HMFG2 monoclonal antibody, which specifically binds to the variable number of tandem repeats (VNTR) region of MUC1, an epitope commonly exposed on tumor cells due to altered glycosylation^26–28^. These second-generation CAR MUC1 T cells were previously validated and shown to exert potent cytotoxic function against breast cancer cells^24^. However, in CCA, CD274 (PD-L1) was concurrently upregulated in tumor tissue, indicative of an immunosuppressive TME that could limit CAR-T cell efficacy through PD-1/PD-L1-mediated exhaustion^29,30^. Anti-PD-1 antibodies, including nivolumab and pembrolizumab, have demonstrated efficacy in the treatment of various solid tumors^31^. Additionally, the paracrine secretion of an anti-PD-1 scFv derived from pembrolizumab in the TME resulted in an enhanced antitumor response compared to conventional CAR-T cells combined with systemic anti-PD-1 monoclonal antibody administration, due to localized delivery^32^. However, a previous study demonstrated that an anti-PD-1 scFv derived from nivolumab bound to PD-1 protein but did not inhibit PD-1/PD-L1 interaction in a cell-based assay, likely due to low solubility in tissue culture media^33^.

In this study, to improve the efficacy of MUC1 CAR-T cells in CCA, we incorporated a secreted anti-PD-1 Ig derived from nivolumab to provide localized checkpoint blockade at the tumor site. The addition of anti-PD-1 Ig did not affect MUC1 CAR transgene expression, CAR-T cell fold expansion, or the immunophenotype of CAR-T products. A full-length Ig was used in this study to take advantage of its intact Fc region, which enables recycling via the neonatal Fc receptor (FcRn) pathway^34–36^, thereby supporting sustained in vivo kinetic and prolonged therapeutic effects^37^. Interestingly, we observed a significant reduction in surface PD-1 expression on MUC1.PD1 CAR-T cells following repeated antigen stimulation, compared to conventional MUC1 CAR-T cells. Previous studies have suggested that such a decrease may reflect transcriptional downregulation of PD-1 mediated by autocrine secretion of anti–PD-1 immunoglobulin^38,39^. However, in our study, PDCD1 transcript levels were not reduced in MUC1.PD1 CAR-T cells after repeated antigen stimulation compared with MUC1 CAR-T cells. Thus, it is more likely that the secreted anti-PD-1 antibody competitively blocks the epitope recognized by the detection antibody in flow cytometry, thus masking PD-1 surface expression without altering actual PD-1 protein levels^40^. Previous studies have reported similar findings where CAR-T cells secreting PD-1 or PD-L1 blocking antibodies exhibited reduced detectable PD-1 expression on their surface, which may result from either receptor downregulation or steric hindrance by the secreted antibody^41,42^. Furthermore, we demonstrated that MUC1-specific CAR-T cells engineered to secrete anti-PD-1 Ig exhibit superior antitumor activity in a xenograft murine model of cholangiocarcinoma (CCA), compared to conventional MUC1 CAR-T cells. While both CAR-T cell types were equally effective in mediating cytotoxicity in vitro, only the MUC1.PD1 CAR-T cells significantly delayed tumor growth in vivo. These findings underscore a common limitation in CAR-T cell therapy for solid tumors, where antigen targeting alone is often insufficient due to the highly immunosuppressive tumor microenvironment (TME)^41,43^. Unlike prior studies using NSG (NOD.Cg-PrkdcscidIl2rgtm1Wjl/SzJ) mice, which lack T, B, and NK cells and exhibit profound innate immune defects^44^, our in vivo experiments were conducted in NOD/SCID mice. These mice lack adaptive immunity but retain partial innate immune function, including NK cell activity, macrophages, dendritic cells, and complement, making human T cell engraftment more challenging^45^. Previous studies have shown that NSG mice support more efficient human T cell engraftment compared to NOD/SCID models^46^. In our NOD/SCID model, conventional second-generation MUC1 CAR-T cells, despite strong in vitro cytotoxicity, failed to mediate significant tumor control likely due to residual innate immune barriers limiting T cell engraftment, expansion, and persistence. By contrast, MUC1 CAR-T cells engineered to secrete anti-PD‑1 immunoglobulin achieved substantial tumor suppression under the same conditions, highlighting their enhanced antitumor activities.

To inhibit the PD-1/PDL-1 signal, alternative strategies have also been explored. Intrinsic knockout of the PDCD1 gene in CAR-T cells has been widely investigated and has been shown to restore T-cell function and enhance antitumor activity^47,48^. In contrast, our study employed a strategy in which CAR-T cells were engineered to locally secrete anti–PD-1 Ig within the tumor microenvironment. This paracrine approach may offer some potential advantages, including support bystander T cells infiltrating the tumor, while potentially minimizing systemic immune-related adverse effects associated with systemic PD-1 blockade. Nevertheless, further studies using immunocompetent animal models are warranted to evaluate bystander effects, characterize tumor-infiltrating lymphocytes (TILs), and define the underlying mechanisms. In addition, direct comparison with standard CAR-T cells combined with systemically delivered anti–PD-1 antibody would be important to determine the relative therapeutic benefit and clinical relevance of this strategy. Finally, our study focuses on PD-1 blockades to enhanced CAR-T cell function. However, other immune checkpoint molecules, TIM-3, LAG-3, and CTLA-4 also play critical roles in T cell exhaustion and may limit the efficacy of PD-1 inhibition alone. These additional inhibitory pathways can impair CAR-T cell persistence and diminish antitumor responses^49,50^. Consequently, combination therapies targeting multiple checkpoints may offer a more comprehensive approach to augment CAR-T cell efficacy. Indeed, previous studies have shown that simultaneous blockade of several inhibitory receptors can sustain T cell activity and improve tumor eradication^50–52^. Further investigation is needed to explore these combination strategies and optimize their integration into CAR-T cell therapeutic regimens.

In summary, the presence of anti-PD-1 Ig significantly maintains the proliferative capacity of MUC1 CAR-T cells in an in vitro repeated antigen stimulation experiment and enhances antitumor efficacy *in vivo.* These results highlight the promise of secreting immune modulators within the TME as an effective strategy to improve antitumor activities of CAR-T cells in cholangiocarcinoma.

## Electronic Supplementary Material

Below is the link to the electronic supplementary material.

Supplementary Material 1

Supplementary Material 2

## Data Availability

The authors confirm that the data supporting the findings of this study are available within the article and its supplementary materials.
