# Supplementary material for: MUC1-targeted CAR-T cell secreted anti-PD-1 IgG antibody enhances antitumor activity in Cholangiocarcinoma
Source: Sci Rep. 2026 Apr 21;16:18597. doi: 10.1038/s41598-026-49988-w (PMC13270150; doi:10.1038/s41598-026-49988-w)
Supplement: Supplementary file 1 — Supplementary Material 1 [file 41598_2026_49988_MOESM1_ESM.docx]

| Reagent or antibody | Source | Cat no. |
| --- | --- | --- |
| PerCP-Cy™ 5.5 Mouse Anti-Human CD3; clone UCHT1 | BD Biosciences | Cat# 560835 |
| Phycoerythrin (PE) Mouse Anti-Human CD8; clone SK1 | BD Biosciences | Cat# 340046 |
| FITC mouse IgM, κ Isotype Control; clone G155228 | BD Biosciences | Cat# 564680 |
| FITC anti-human TIM-3 (CD366); clone 7D3 | BD Biosciences | Cat# 565568 |
| VioBlue mouse IgG2b, κ Isotype Control; clone 27-35 | BD Biosciences | Cat# 562748 |
| VioBlue anti-human LAG-3 (CD223); clone T47-530 | BD Biosciences | Cat# 565720 |
| VioGreen IgG2a, κ Isotype Control; clone MOPC-173 | BD Biosciences | Cat# 563483 |
| VioGreen anti-human TIGIT; clone 741182 | BD Biosciences | Cat# 747482 |
| PE-Cy™7 Mouse IgG1 κ Isotype Control; clone MOPC-21 | BD Biosciences | Cat# 565573 |
| PE-Cy™7 anti-human CD279 (PD-1); clone EH12.1 | BD Biosciences | Cat# 561272 |
| AF-488 anti-human IgG (H+L) | Jackson ImmunoResearch | Cat#109-607-003 |
| Phycoerythrin (PE) Mouse Anti-Human CD3; Clone UCHT1 | BD Biosciences | Cat# 555333 |
| APC anti-human CD19 | BD Biosciences | Cat#561742 |
| VioGreen anti human CD45RO; clone UCHL1 | Biolegend | Cat# 304246 |
| VioBlue anti human CD62L; clone  DREG-56 | Biolegend | Cat# 304828 |

Supplementary Table 1. Monoclonal Antibodies Used for Flow Cytometry Analysis
