# Supplementary material for: MUC1-targeted CAR-T cell secreted anti-PD-1 IgG antibody enhances antitumor activity in Cholangiocarcinoma
Source: Sci Rep. 2026 Apr 21;16:18597. doi: 10.1038/s41598-026-49988-w (PMC13270150; doi:10.1038/s41598-026-49988-w)
Supplement: Supplementary file 2 — Supplementary Material 2 [file 41598_2026_49988_MOESM2_ESM.docx]

**MUC1-targeted CAR-T cell secreted anti-PD-1 IgG antibody enhances antitumor activity in Cholangiocarcinoma**

Nattarika Khuisangeam^1,2^, Thanyavi Chinsuwan^2,3^, Thananya Intanachai^1,2^, Rattapoom Thaiwong^2,4^, Chatikorn Boonkrai^5^, Tanapati Phakham^5^, Trairak Pisitkun^5^, Koramit Suppipat^2,6^, Nattiya Hirankarn^7^, Supannikar Tawinwung^2,8*^

**Supplement figure 1.**


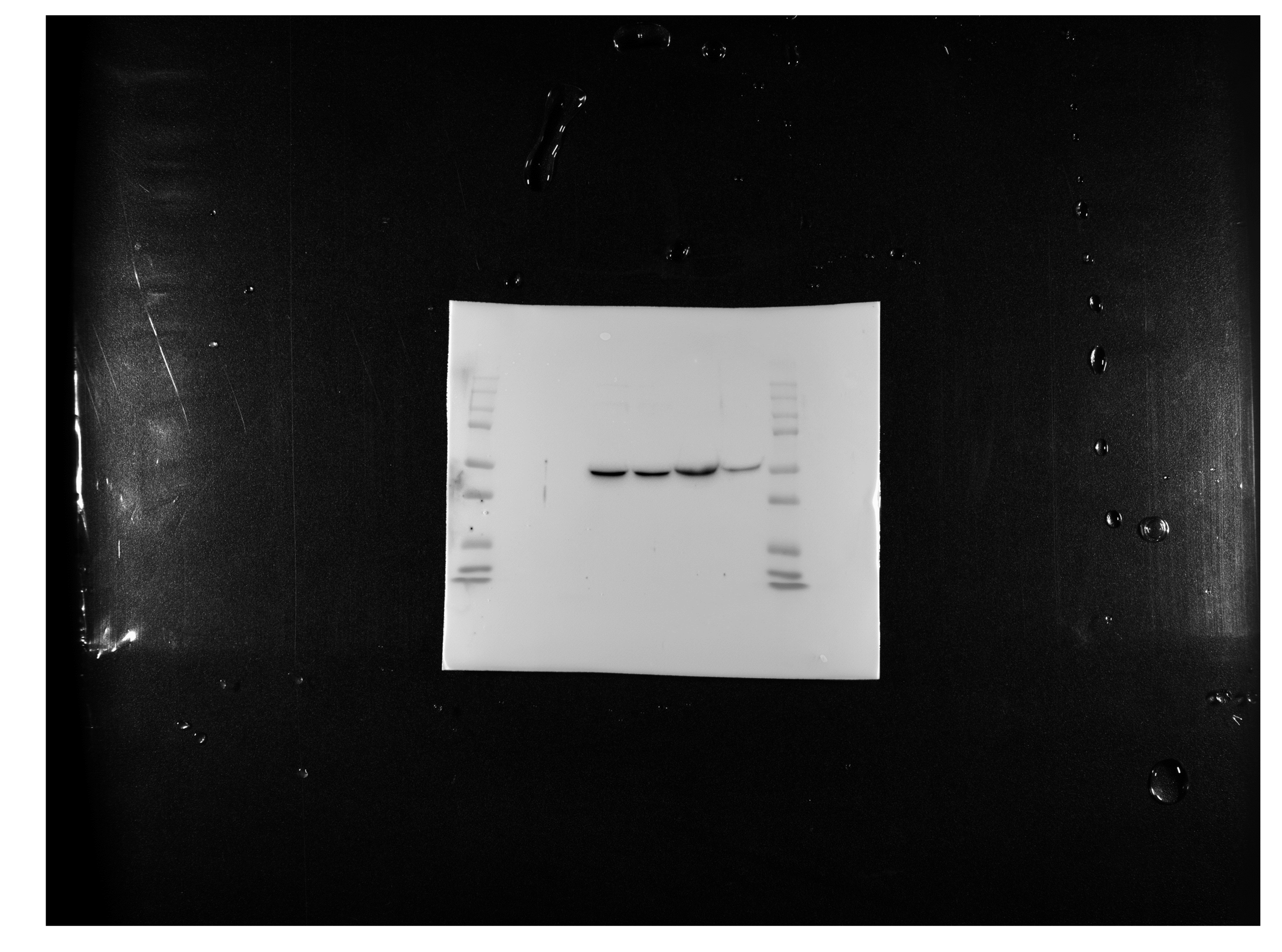


**Figure S1. Uncropped image of the Western blot membrane used for Figure 2D.**

**Supplement Figure 2**

**
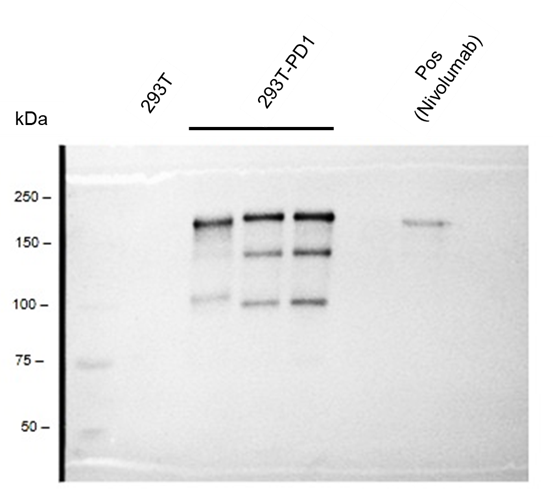
**

**Figure S2. Validation of recombinant anti-PD-1 antibody secreted from 293T cells.** Secreted anti-PD-1 antibodies were analyzed by Western blot using an HRP-conjugated goat anti-human Fc antibody. Under non-reducing conditions, a predominant band was observed at approximately 150 kDa, corresponding to the fully assembled IgG heterotetramer (H₂L₂), comparable to the positive control (nivolumab). Additional bands at approximately 125 kDa and 100 kDa likely represent partially assembled species (H₂L₁) and heavy-chain homodimers (H₂), respectively.

**Supplementary figure 3.**


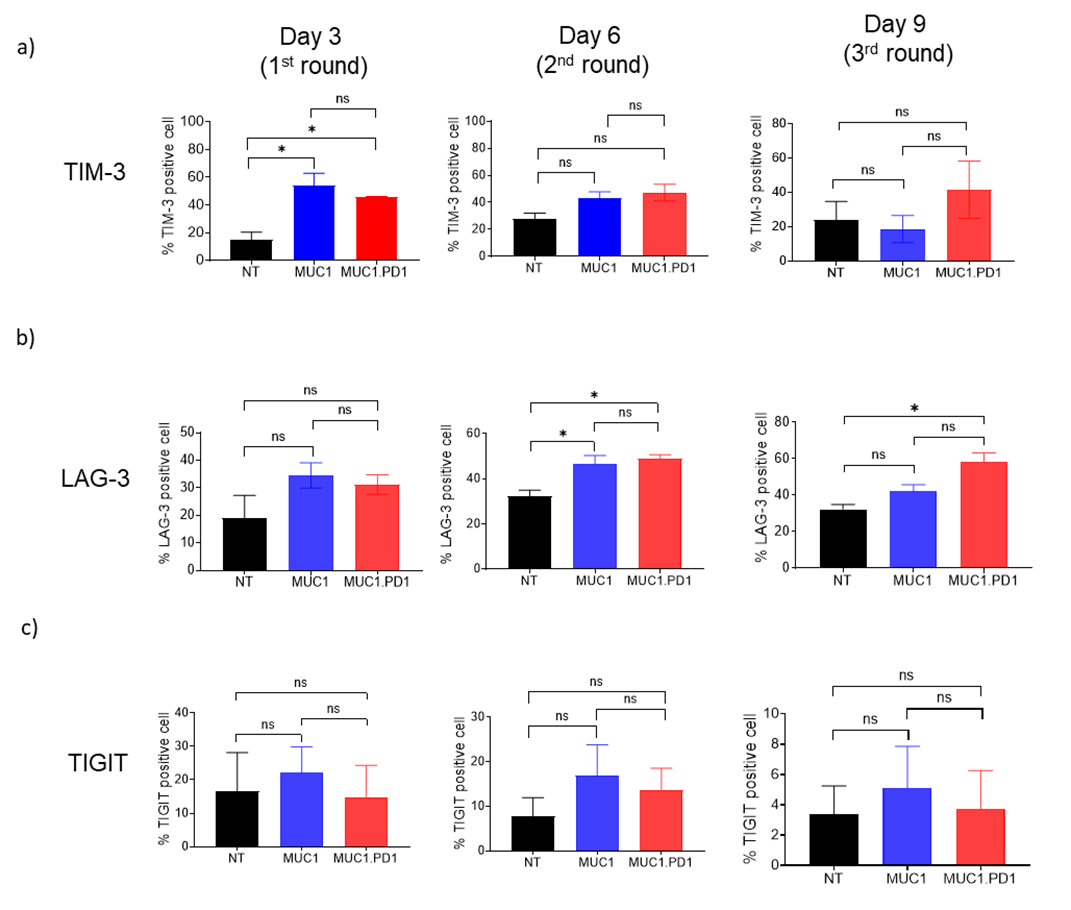


**Figure S3.** **Expression of additional T cell exhaustion markers following repeated antigen stimulation with MUC1⁺/PD-L1⁺ CCA cells.** Surface expression of exhaustion markers was assessed by flow cytometry at the end of each stimulation round. The bar graphs show the mean percentage of T cells expressing (a) TIM-3, (b) LAG-3, and (c) TIGIT following each round of co-culture. All data are presented as mean ± S.E.M. (n = 3 donors/group). Statistical significance for surface exhaustion markers comparisons was determined by one-way ANOVA with Tukey’s multiple comparisons test. ANOVA, analysis of variance; ns = not significant. *p < 0.05.

**Supplementary figure 4.**

**
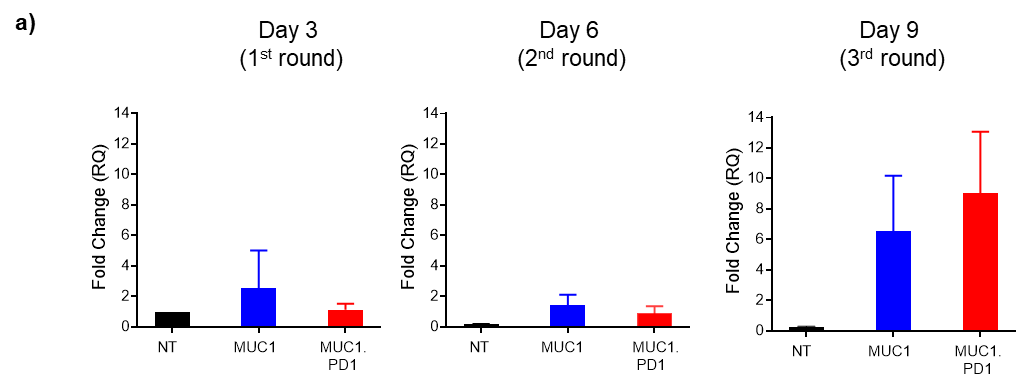
**

**Figure S4.** PDCD1 transcript levels during repeated antigen stimulation with HuCCT-1 cells. PDCD1 mRNA expression was assessed across three rounds of co-culture of MUC1 CAR-T, MUC1.PD1 CAR-T, and non-transduced (NT) T cells with the CCA cell line HuCCT-1. CAR-T cells were re-challenged with fresh HuCCT-1 cells every 72 hours. Total RNA was isolated at the end of each round and reverse-transcribed into cDNA. PDCD1 transcript levels were quantified by qPCR using Luna® Universal SYBR Green qPCR Master Mix (New England Biolabs). Expression was normalized to GAPDH and calculated as fold change (RQ) relative to NT. No statistically significant differences in PDCD1 expression were observed between MUC1 CAR-T, and MUC1.PD1 CAR-T within any individual round (p > 0.05). Data represent mean ± S.E.M. (n = 3). Statistical analysis was performed using one-way ANOVA with Tukey’s multiple comparisons test.
